# DietPal: A Web-Based Dietary Menu-Generating and Management System

**DOI:** 10.2196/jmir.6.1.e4

**Published:** 2004-01-30

**Authors:** Shahrul A Noah, Siti Norulhuda Abdullah, Suzana Shahar, Helmi Abdul-Hamid, Nurkahirizan Khairudin, Mohamed Yusoff, Rafidah Ghazali, Nooraini Mohd-Yusoff, Nik Shanita Shafii, Zaharah Abdul-Manaf

**Affiliations:** ^1^Faculty of Information Science & TechnologyUniversiti Kebangsaan Malaysia43600 UKM, Bangi Selangor Darul EhsanMalaysia; ^2^Department of Nutrition & DieteticsFaculty of Allied Health SciencesUniversiti Kebangsaan MalaysiaJalan Raja Muda Abdul Aziz 50300 Kuala LumpurMalaysia

**Keywords:** Dietary services, menu planning, health, information systems, health information system, Web-based services, Internet

## Abstract

**Background:**

Attempts in current health care practice to make health care more accessible, effective, and efficient through the use of information technology could include implementation of computer-based dietary menu generation. While several of such systems already exist, their focus is mainly to assist healthy individuals calculate their calorie intake and to help monitor the selection of menus based upon a prespecified calorie value. Although these prove to be helpful in some ways, they are not suitable for monitoring, planning, and managing patients' dietary needs and requirements. This paper presents a Web-based application that simulates the process of menu suggestions according to a standard practice employed by dietitians.

**Objective:**

To model the workflow of dietitians and to develop, based on this workflow, a Web-based system for dietary menu generation and management. The system is aimed to be used by dietitians or by medical professionals of health centers in rural areas where there are no designated qualified dietitians.

**Methods:**

First, a user-needs study was conducted among dietitians in Malaysia. The first survey of 93 dietitians (with 52 responding) was an assessment of information needed for dietary management and evaluation of compliance towards a dietary regime. The second study consisted of ethnographic observation and semi-structured interviews with 14 dietitians in order to identify the workflow of a menu-suggestion process. We subsequently designed and developed a Web-based dietary menu generation and management system called DietPal. DietPal has the capability of automatically calculating the nutrient and calorie intake of each patient based on the dietary recall as well as generating suitable diet and menu plans according to the calorie and nutrient requirement of the patient, calculated from anthropometric measurements. The system also allows reusing stored or predefined menus for other patients with similar health and nutrient requirements.

**Results:**

We modeled the workflow of menu-suggestion activity currently adhered to by dietitians in Malaysia. Based on this workflow, a Web-based system was developed. Initial post evaluation among 10 dietitians indicates that they are comfortable with the organization of the modules and information.

**Conclusions:**

The system has the potential of enhancing the quality of services with the provision of standard and healthy menu plans and at the same time increasing outreach, particularly to rural areas. With its potential capability of optimizing the time spent by dietitians to plan suitable menus, more quality time could be spent delivering nutrition education to the patients.

## Introduction

Planning nutritious and appetizing menus is a complex task that researchers have tried to computerize since the early 1960s [1,2]. Although a number of menu-planning systems have been developed in recent years, these systems are mainly used to assist healthy individuals calculate their calorie intake and to help monitor the selection of menus based upon a prespecified calorie value. Also, some of these systems do not address the standard practice and procedure employed by dietitians during consultations with patients. Planning nutritious menus for patients, however, is not the same as planning menus for healthy individuals. Patients require advice and directions from dietitians in designing their menus. Dietitians on the other hand, during the course of consultation with a patient, may want to refer, for example, to the patient's medical and dietary history, dietary recall, biochemical data, and anthropometric data. to construct a suitable dietary plan and menu for patients. For example, a suitable menu for a diabetic patient is constructed based on the patient's calorie requirement determined using anthropometric data such as weight and height. The patient's blood sugar control is examined to decide on refined-sugar allowances. Finally, obtaining the dietary history of a particular patient is necessary in order to consider factors such as food habit and preferences. Therefore, an ideal menu-planning system should not only contain information about foods and menus but should also incorporate other related information for the purpose of decision making by dietitians (as discussed earlier).

Realizing the limited capabilities of existing systems, this paper, therefore, describes the development of an automated Web-based menu-generating system, according to a standard procedure and practice adhered to by dietitians in managing patients, and based upon a user-needs study conducted prior to the development of the system. This project is a collaboration between the Faculty of Information Science and Technology and the Faculty of Allied Health Sciences (see "Acknowledgements" for funding information).

At the moment, the system is intended for use by dietitians and health professionals to extract patients' dietary recalls and to design suitable menus based on a patient's dietary habits and nutritional requirements.

### Related Research and Development

As previously mentioned, there have been a number of menu-generating systems or dietary-analysis programs available, either implemented as a Web-based application [3- 8] or as a traditional information system [9- 11].

A few systems reviewed—such as Case-Based Menu Planner (CAMP) [12], Pattern Regulator for the Intelligent Selection of Menus (PRISM) [13], and CAMP Enhanced by Rules (CAMPER) [11]—employed techniques from the field of artificial intelligence (AI):

- CAMP employs the case-based reasoning (CBR) technique to suggest menus to users. CAMP uses past menus that were compiled from reputable sources and modified as needed to ensure that they satisfy the RDIs (Reference Daily Intakes) and the Dietary Guidelines of Americans and Aesthetic standards [10]. The menu generated by CAMP is based upon nutrient composition, type of servings, and the number of snacks.
- PRISM uses rules to generate menus. The rules are mainly concerned with menus and meal patterns.
- CAMPER is an integration of the techniques employed by CAMP and PRISM. Therefore, apart from using the case-based reasoning technique, CAMPER uses rules or "what if" analysis module to enhance the menu suggestion activity.

The main interesting and distinguishing feature exhibited by our system as compared to the other reviewed systems is its use of the complete dietary-management system currently adhered to by dietitians in Malaysia, particularly at the National University Hospital of Malaysia. In addition, our system exploits current advanced Internet technology, by considering the system's implementation as a Web-based application. This, to a certain extent, increases the outreach of the system for use by dietitians and health professionals within the same hospital or at other locations. The system is also capable of storing and organizing patients' dietary records and other health-diet related information. This capability would allow dietitians to effectively evaluate or monitor the patients' dietary changes throughout the period of consultations.

## Methods

### User Information Needs Study and Functional Specification

Prior to the development of the system, a user-needs study was conducted among dietitians within the Klang Valley in Malaysia [14].. The user-needs study included 2 independent surveys. The first survey of dietitians was on computer literacy and utilization, information seeking activities, and assessment of information required for dietary management and evaluation of compliance towards a dietary regime. The second survey consisted of semistructured interviews with a subsample of dietitians in order to extract the knowledge and workflow of a menu-suggestion process.

### Development of DietPal

DietPal was developed as a Web-based system in order to increase outreach, particularly in rural areas. The main scripting language used is Active Server Pages (ASP) together with other scripting languages, mainly VBScript and JavaScript.

The development of DietPal took into account the key findings of the user-needs study and the consultation flow currently adhered to by dietitians.

### Post-evaluation

An initial post-evaluation has been conducted by distributing a written survey among 10 dietitians who are directly involved in the management of patients from the National University of Malaysia Hospital.

## Results

### User Information Needs Study and Functional Specification

In the first survey, questionnaires were posted to 93 clinical dietitians registered with the Malaysia Dietitians' Association; 52 subjects (56%) responded. The questionnaire contained the question "Which information items do you seek to support decision making and evaluation of a dietary regime", with the 10 items listed in Table 1 and participants asked to assign a rating score of 1 to 5 (1 = least likely seek, 5 = most likely seek) was used to identify the information needed to. In a similar way, participants were asked which information items they needed in order to monitor the compliance of a patient (Table 2).

Table 1 and Table 2 present part of the results obtained from this user-needs study. Because the questionnaire was self administered not all items were responded to by all 52 subjects; thus, missing data was unavoidable.

**Table 1:** Results of the information-needs survey of 52 dietitians

**Information Need**	Mean Score ± SD*
Medical diagnosis	4.80 ± 0.45
Current body height and weight	4.69 ± 0.65
Nutrient requirement	4.57 ± 0.88
Biochemical values	4.55 ± 0.86
Dietary recall	4.53 ± 0.86
Weight	4.18 ± 1.01
Medical history†	4.14 ± 0.95
Medication	3.71 ± 1.15
Allergy	3.35 ± 1.32
Supplement	3.31 ± 1.24

^*^ Frequency score: 1= least likely seek, 5=most likely seek.

^†^ N = 50.

**Table 2:** Results of the indicators for patient compliance survey

Data Type*	**N**	**Mean Score ± SD**
Biochemical values	51	4.69 ± 0.62
Food intake change	52	4.63 ± 0.60
Compliance to diet regime	52	4.62 ± 0.57
Weight change	51	4.61 ± 0.57
Clinical parameters	49	4.53 ± 0.65
Changes of knowledge/behavior	52	3.98 ± 1.09
Physical activities	50	3.76 ± 1.13
Changes of medication	50	3.14 ± 1.25

^*^ Frequency score: 1=most frequent, 5=least frequent.

Based upon the results presented in Table 1 and Table 2, the most common information used to support decision making according to the mean of scores were medical diagnosis, current body weight and height, nutrient requirement, biochemical values, and dietary recall.

The most relevant indicators for monitoring patient compliance were reported to be biochemical values, food intake changes, compliance to diet regime, and weight change.

**Figure 1:**
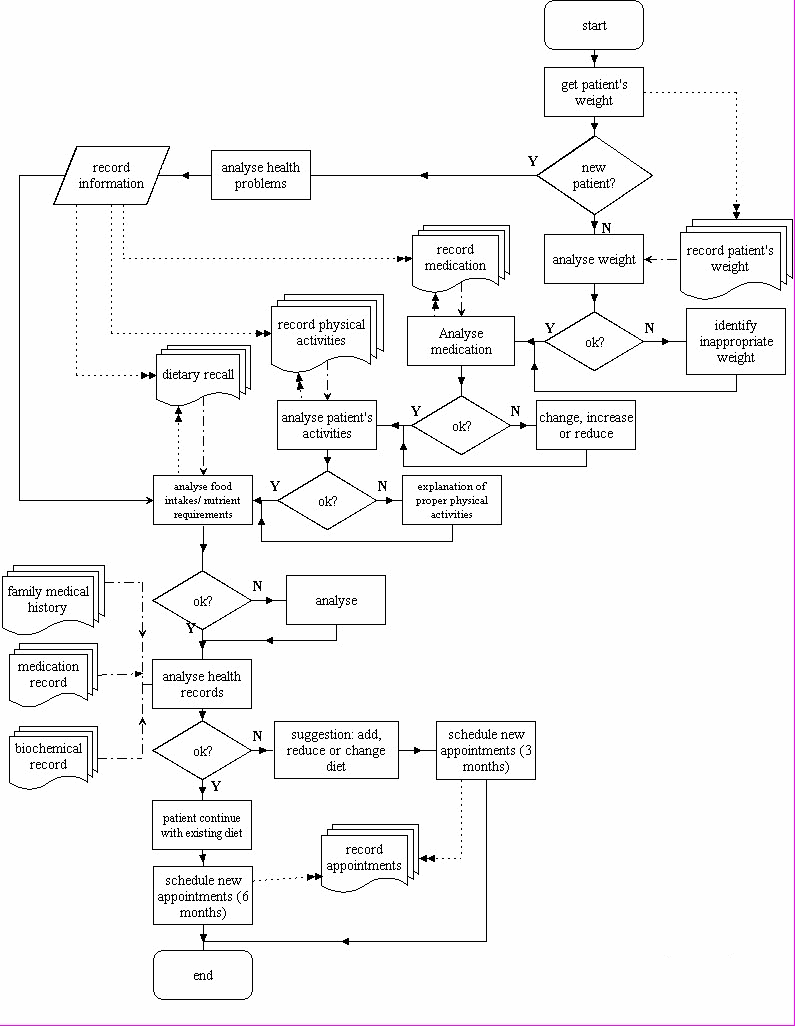
The workflow of menu-suggestion activity, as determined by observing and interviewing dietitians in Malaysia. A solid line with single arrowhead refers to flow of menu-suggestion activity. A dotted line with double arrowhead refers to the process of recording information or moving a record to the respective forms or data repository. A dashed-plus-dotted line with single arrowhead refers to the process of retrieving previous/existing data

The second survey involved ethnographic observation of 14 clinical dietitians from government, university hospitals, and private hospitals doing individual dietary counseling. This study was similar to an information-engineering activity, in which the task was to extract the knowledge and the workflow of generating a menu for patients. In this case, 14 dietitians were interviewed and observed while conducting their daily activities. The survey was also aimed at validating the results of the first survey and determining at which stage of the menu-suggestion process the "frequently sought after" information items are being used. The result of this survey is a workflow of a menu-suggestion process currently used by dietitians in Malaysia, as illustrated in Figure 1.

### Menu Generation and Management with DietPal

**Figure 2:**
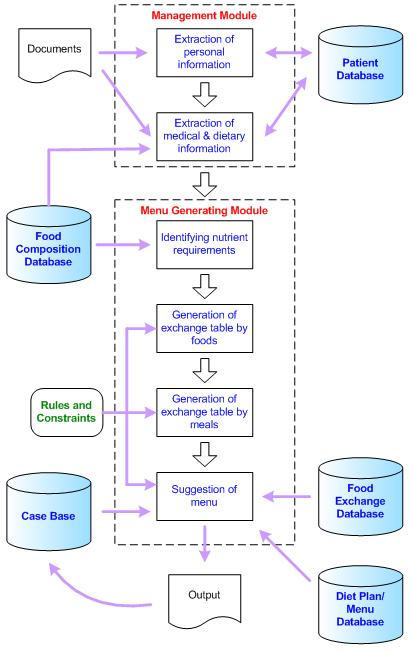
The DietPal system architecture

As illustrated in DietPal's system architecture (Figure 2), the system consists of 5 databases with 2 main modules, the Management module and the Menu Generating Module. The Management module is designed to manage information relating to patients' personal and medical information and to assess patients' compliance to dietary regime. The Menu Generating module is designed to interactively assist users in planning suitable menus and diet plans for patients. The Patient database is used to store the patient's information, which includes the personal data, anthropometric and biochemical data, medical record, and information on dietary recall. This database is heavily used in the Management module. The Food Composition database consists of information about foods and nutrient composition, extracted from the Malaysian Food Composition Tables for macronutrients and micronutrients. This database is used for food-analysis purposes. The Diet Plan/Menu database consists of therapeutic menus for specific diseases; the menus were obtained from reputable sources and have been approved by dietitians.

When using DietPal for an existing patient, the dietitian is first required to update the anthropometric data, particularly the body weight; this is to record any important patient changes since the previous meeting. If current biochemical data is available, such data will also be recorded by the dietitian; this is to monitor the patient progress so as to evaluate patient compliance to the previously-suggested menus. Information regarding any new medical diagnosis and current medication, if any, will also be recorded. For new patients, the dietitian is required to formally register the patients with the system; information such as name, date of birth, address, occupation, and other information similar to that for existing patients will be recorded.

The next step involves acquiring the patient's dietary recall in order to assess the patient's compliance to a dietary regime as well as to assist dietitians in planning suitable menus based on the patient's food habits. In normal circumstances, the dietitian will record the patient dietary recall of up to 7 days of food intake. In this case, the dietitian will interview the patient and will select the available food stored in the food-composition database. For cases in which the food is not available in the database, the dietitian will choose other similar types of food based on calorie content. DietPal will automatically calculate the level of macronutrients and micronutrients for an average of 1 day. Apart from that, DietPal also allows the user to compare the level of macronutrients with the individual calorie requirement based on Basal Metabolic Rate (BMR), physical activity, and stress factor, while the micronutrient intake is compared with the Malaysian Recommended Dietary Allowance (RDA) [15] for nutrients determined according to the patient's sex and age group. The task of dietary recall is important for assessing the patient's compliance to the dietary regime, but, because this process is time consuming, it can be passed over by the dietitian, who can go directly to the menu-generating function.

The menu generation starts with DietPal automatically calculating the Body Mass Index (BMI) and providing a suggestion as to whether the patient is, for example, normal, obese, or underweight. Simple rules are used to make such a decision. Anthropometric information is also displayed by DietPal in order to assist dietitians in making decisions. Two methods can be used to automatically generate the energy requirements of each individual patient:

- Predictive equation to estimate energy requirement based on Basal Metabolic Rate (BMR) (Table 3), stress factor (Table 4), and activity factor (Table 5). The energy requirement is estimated using the following formula [16]:
  Energy requirement = BMR × stress factor × activity factor.
- Quick method, based on the following formula:
  Energy requirement = weight (kg) × quick method factor (kcal/kg)

Values of the quick method factor based on weight status and physical activity are shown in Table 6 [17].

**Table 3:** Equations to predict Basal Metabolic Rate (BMR)

**Age Range** **(Years)**	**Equation for** Men*	**Equation for** Women*
15-18	17.6W + 656	13.3W + 690
18-30	15.0W + 690	14.8W + 485
30-60	11.4W + 870	8.1W + 842
> 60	11.7W + 585	9.0W + 656

^*^ W = weight (kg)

**Table 4:** Stress factor in clinical situation

**Clinical Situation**	**Stress Factor**
Starvation	0.85
Elective surgery	1.05-1.15
Sepsis	1.20-1.40
Head injury	1.30
Trauma	1.40
Inflammation	1.50
Burns	2.0

**Table 5:** Activity factor

**Activity**	**Activity Factor**
Bed bound immobile	1.0-1.2
Out of bed	1.3-2.0

**Table 6:** Quick method factor

**Physical Activity**	**Quick Method Factor**		
	**Overweight**	**Normal Weight**	**Underweight**
Sedentary	20-25 kcal/kg weight	30 kcal/kg weight	35 kcal/kg weight
Moderate	20-25 kcal/kg weight	35 kcal/kg weight	40 kcal/kg weight
Marked	20-25 kcal/kg weight	40 kcal/kg weight	45 kcal/kg weight

**Figure 3:**
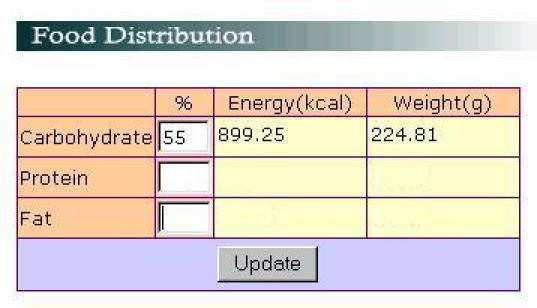
An interface allowing dietitians to edit the contributions of macronutrients

Once the energy requirement has been determined, the macronutrient requirement will be calculated (the energy is distributed into 3 macronutrients, see Table 7). In this case, the dietitian will provide the percentage of carbohydrate, protein, and fat (C-P-F) and DietPal will automatically calculate the kilocalories and grams of carbohydrate, protein, and fat according to the energy requirement of the particular patient. Values in Table 7 are the default values provided by the system; they are derived from the Malaysian Dietary Guidelines [18]. Dietitians, however, are allowed to alter these values based on patients' requirement as illustrated in Figure 3. If the total percentage of all nutrients exceeds 100% or is below 100% an error message is provided to the user. Based on these nutrient percentages, the kilocalories and grams of the nutrient are automatically generated by the system.

**Table 7:** Contribution of macronutrients to total calories

**Nutrient**	Percentage of Total Calories*
Carbohydrate	55
Protein	15
Fat	30

^*^ Default values provided by the system; derived from Malaysian Dietary Guidelines.

The next step requires the dietitian to design the exchange table by food groups and by meals. An exchange table describes the portions of each food group based on a prefixed amount of carbohydrate, protein, and fat. For example, 1 exchange of food from the cereals food group contains 15 g of carbohydrate, 2.0 g of protein, and 0.5 g of fat. In this case, DietPal will first suggest an exchange table based on the standard calculation currently practiced by Malaysian dietitians. The user is allowed to alter any exchange portions according to the patient's needs; DietPal will update the amount of carbohydrate, protein, fat, and energy accordingly. Once the exchange table by food group has been successfully produced, the dietitian will continue with the design of an exchange table by meal. In this case the dietitian is required to fill in the exchange portions for the relevant mealtimes. DietPal will detect any inconsistency that might occur, such as if the distributed amount of exchange portions is not equal to the total exchanges.

**Figure 4:**
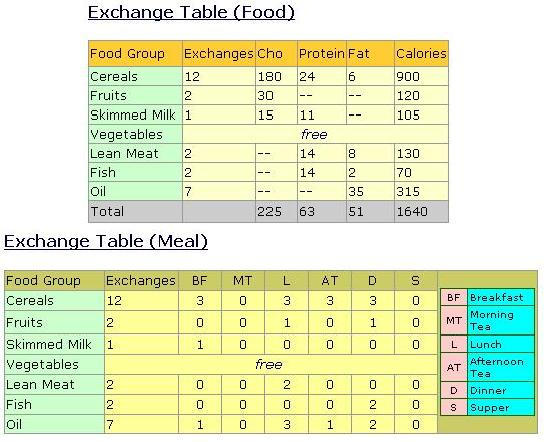
Exchange table by food group and by meal for 1600 kcal/day

As an alternative to the aforementioned process, the dietitian can retrieve the standard existing exchange table by food and by meal stored in the Diet Plan database. To date, DietPal has a number of diet plans ranging from 1200 kcal to 2000 kcal designed by dietitians from the Faculty of Allied Health Sciences of the National University Hospital of Malaysia. Figure 4 illustrates the output of the exchange table by food and by meal for the 1600 kcal standard diet plan. The distribution of the macronutrients are based on a prefixed range of exchanges of each food group (ie, cereal, 8-14 exchanges; fruits, 2 exchanges; skim milk, 1-2 exchanges; vegetables, minimum 2 servings; meat, 1-2 exchanges; fish, 2 exchanges; and oil, 6-10 exchanges). The use of existing diet plans greatly reduced the time for designing such food-distribution tables.

**Figure 5:**
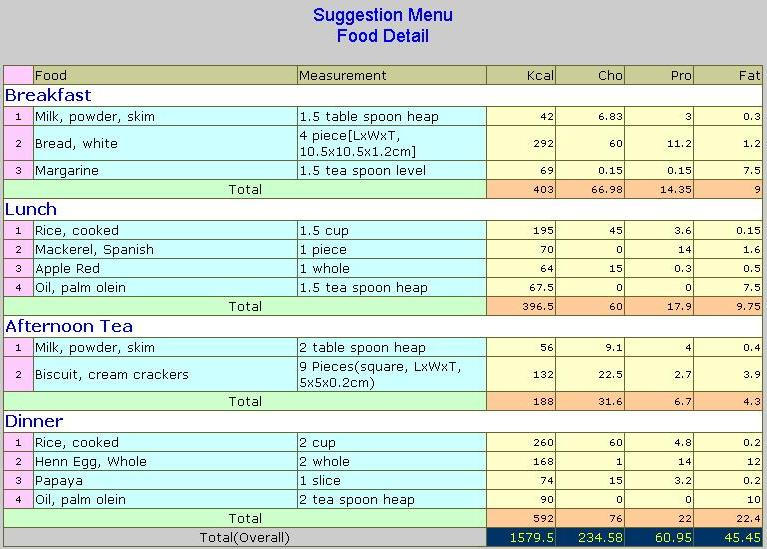
An example of menu/diet plan for 1600 kcal/day designed by using DietPal. Cho refers to carbohydrate and Pro refers to protein

The menu suggestion will be made after all the aforementioned steps have been completed. There are currently 2 available approaches offered by DietPal—either dietitians design the menu based upon the nutrient requirement and exchange tables or they can retrieve and reuse preexisting menus from the Case Base. From the exchange allowances of each food group (Figure 4), the dietitian will then be able to generate a suitable menu based on the Malaysian Food Composition Table. Alternatively, the dietitian can retrieve preexisting menus based upon the nutrient requirement and the disease state of the patient. The retrieved menu can be edited or altered by dietitians according to the suitability of the patient. The output menu—either manually-designed or retrieved from the Case Base—will be stored in the Case Base for future use. Dietitians can design or retrieve more than 1 menu for the patient. Figure 5 illustrates an example of a menu plan generated using DietPal.

Medical professionals of health centers in rural areas where there are no designated qualified dietitians will find the preexisting menus useful for advising patients with certain disease problems. The menu designed is only suitable for nonvegetarians, as vegetarianism is not prominent within the Malaysian society.

**Figure 6:**
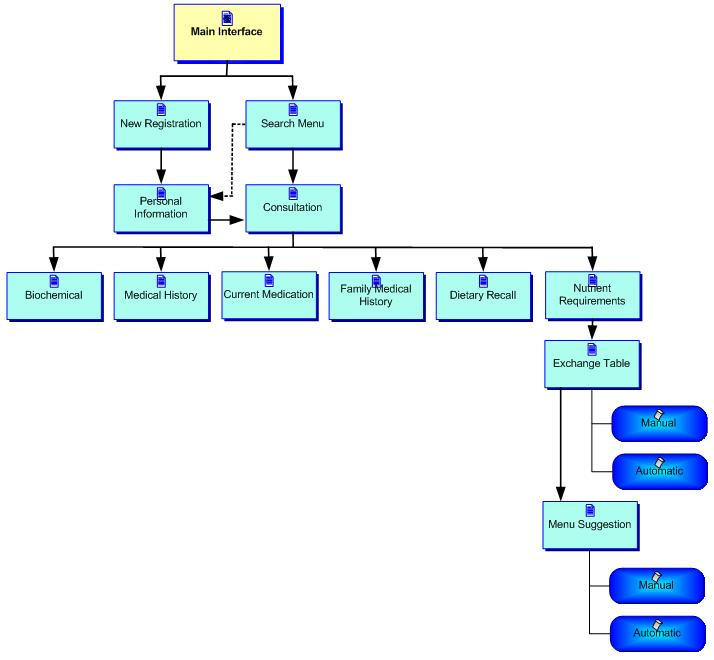
Multiple levels of menus in DietPal

The functionalities of DietPal are distributed to a number of different levels of menus (pages) as illustrated in Figure 6, which correspond to the menu-suggestion activities previously described.

**Table 8:** Results of the post evaluation among 10 dietitians from the National University of Malaysia Hospital

**Category**	Ratings by 10 Dietitians*										
	**R1**	**R2**	**R3**	**R4**	**R5**	**R6**	**R7**	**R8**	**R9**	**R10**	**Average**
1. System usability											
Personal data	4	5	2	3	4	4	5	4	3	5	3.9
Consultation	3	5	3	3	4	4	5	4	4	5	4.0
Biochemical	3	5	2	4	4	4	3	4	4	5	3.8
Medical history	3	2	2	4	4	4	4	4	4	5	3.6
Family medical history	4	5	2	3	4	4	4	4	4	-	3.8
Current medication	4	5	3	3	4	3	3	4	4	5	3.8
Dietary recall	3	5	3	2	4	4	2	5	3	4	3.5
Nutrient/Energy requirements	4	5	3	4	4	5	5	5	4	5	4.4
Exchange table by foods	4	5	3	3	4	5	5	5	4	5	4.3
Exchange table by meals	4	5	3	3	4	4	5	4	4	5	4.1
Menu suggestion	2	5	2	-	4	3	3	4	4	5	3.6
**Average of system's usability**											**3.9**
2. Organization of modules	4	5	3	-	4	3	4	5	2	5	3.9
3. System efficiency	3	5	2	3	4	5	4	5	3	5	3.9
4. System's accuracy	4	2	3	3	4	5	4	4	3	5	3.7
5. Satisfy user's requirement	4	5	3	4	4	5	4	4	4	5	4.2

^*^ Ratings scale: 1 = strongly disagree/strongly dissatisfied; 2 = disagree/unsatisfied; 3 = average; 4 = satisfied/agree; 5 = strongly agree/strongly satisfied

### Post-evaluation

A preliminary evaluation has been conducted among 10 dietitians who are directly involved in the management of patients from the National University of Malaysia Hospital and who used the system. The result of this evaluation is illustrated in Table 8, where scale = 1 represents strongly disagree/unsatisfied and scale = 5 represents strongly agree/satisfied. The categories used during evaluation were derived from [19- 21].

Results from Table 8 show that the system scores above average for all the categories evaluated, with *satisfy user's requirement* scoring the highest marks. These results indicate that on average dietitians are satisfied with the overall capability of the system to generate and manage dietary menus.

## Discussion

The adoption of Internet- or Web-based technology in health-related applications is still lagging well behind adoption in other fields [22]. It is expected that if communications through the Internet and the World Wide Web are looked into seriously, the efficiency of delivering health care and services could be increased [23].

DietPal is a dietary menu generation and management system for patients, with simple intelligent capabilities to design and generate suitable diet plans and menus based upon the patient's energy requirement. Comparing this system with other similar Web-based systems—such as the Menu Planner developed by the National Health, Lung and Blood Institute (NHLBI) [6]; the Nutrition Analysis Tool (NAT) [7], a public service system provided by the Food Science and Human Nutrition Department at the University of Illinois; and DietSite.com [8], provided by Dietsite.com Inc—the system exhibits a few distinguishing features:

- capacity to store and retrieve historical data related to a patient, which can be used to assess the patient's compliance to the dietary regime
- retrieval of preexisting menus that suit the requirements of an existing patient
- capacity to monitor the progress of each patient based upon the menu suggested

More rigorous testing and evaluation of the system is currently ongoing. Based on the preliminary experiences reported here, we think that it has the potential to provide the following significant contributions:

- Assist in enhancing the quality of health services and improving the outreach in urban and, particularly, rural areas with minimal costs.
- Provide standards for healthy menus for patients. Hospitals and clinics in rural areas in particular, therefore, will be able to access an up-to-date database specifying the needs of certain patients.
- Optimize time spent by dietitians to calculate nutrient intake and energy requirements, and to generate exchange tables and menus. Thus, dietitians will have more time to deliver nutrition education to patients. A cost-effectiveness study on the usage of the system among dietitians or health professionals will be conducted.

Our ongoing work includes applying artificial-intelligence techniques to intelligently generate suitable menus for patients with certain diseases such as diabetes, hyperlipidaemia, obesity, and hypertension. We are exploring the case-based reasoning technique, where a new problem is solved by finding similar past cases, and reusing them in the new problem situation [24]. Although such a technique has been used in CAMP [12] and CAMPER [11], our approach differs from those two in the following aspects:

- We will consider the 4 major diseases (diabetes, hyperlipidaemia, obesity, and hypertension) in generating the required diet plans or menus.
- As Malaysia is a multiracial country (eg, Malay, Chinese, Indian), we need to consider menus according to the respective ethnicities.
- We will consider the patient's dietary recall as well as the patient's dietary exchanges in providing suitable menus.

## Abbreviations

BMR: Basal Metabolic Rate
